# Colletotrichum Gloesporioides Inhibition In Situ by Chitosan-*Ruta graveolens* Essential Oil Coatings: Effect on Microbiological, Physicochemical, and Organoleptic Properties of Guava (*Psidium guajava* L.) during Room Temperature Storage

**DOI:** 10.3390/biom9090399

**Published:** 2019-08-22

**Authors:** Carlos David Grande Tovar, Johannes Delgado-Ospina, Diana Paola Navia Porras, Yeimmy Peralta-Ruiz, Alexander Pérez Cordero, Jorge Iván Castro, Manuel Noé Chaur Valencia, José Hermínsul Mina, Clemencia Chaves López

**Affiliations:** 1 Grupo de Investigación de fotoquímica y fotobiología, Universidad del Atlántico, Carrera 30 Número 8-49, Puerto Colombia 081008, Colombia; 2 Grupo de Investigación Biotecnología, Facultad de Ingeniería, Universidad de San Buenaventura Cali, Carrera 122 # 6-65, Cali 76001, Colombia; 3 Faculty of Bioscience and Technology for Food, Agriculture and Environment, University of Teramo, Via R. Balzarini 1, 64100 Teramo, Italy; 4 Facultad de Ingeniería, Programa de Ingeniería Agroindustrial, Universidad del Atlántico, Carrera 30 Número 8-49, Puerto Colombia 081008, Colombia; 5 Grupo de Investigación en Bioprospección Agropecuarias, Universidad de Sucre, carrera 28 # 5-267, Puerta Roja - Sincelejo (Sucre) 700008, Colombia; 6 Laboratorio SIMERQO, Departamento de Química, Universidad del Valle, Calle 13 No. 100-00, Cali 76001, Colombia; 7 Escuela de Ingeniería de Materiales, Facultad de Ingeniería, Universidad del Valle, Calle 13 No. 100-00, Santiago de Cali 760032, Colombia

**Keywords:** chitosan, *Colletotrichum gloeosporioides*, edible coating, guava, *Ruta graveolens* essential oil

## Abstract

Guava is a fruit appreciated worldwide for its high content of bioactive compounds. However, it is considered a highly perishable fruit, generally attacked by pathogenic species such as the fungi *Colletotrichum gloeosporioides*, which causes anthracnosis. To diminish the losses caused by pathogenic fungi, coatings of chitosan (CS) with *Ruta graveolens* essential oil (RGEO) in different concentrations (0.5, 1.0, 1.5% *v/v*) were applied in situ and their effects on the physical properties and microbiological quality of the guavas were studied. The CS+RGEO coated fruits exhibited better physicochemical behavior and lower microbiological decay as compared to the uncoated guavas, demonstrating the effectiveness of the coatings, especially those with 1.5% of RGEO content. All the fruits coated had greater acceptance and quality than the controls, being more those with essential oil incorporation. In situ investigation of *C. gloesporioides* infection of guavas demonstrated that the CS+RGEO coated guavas showed a high percentage of inhibition in the development of anthracnose lesions. In the present investigation, an alternative method has been proposed to extend the stability of the guavas fruit up to 12 days with application in the food industry.

## 1. Introduction

The most considerable losses of fruits at the post-harvest level are mainly due to microbial infection during the supply chain, affecting producers and consumers [1,2,3,4,5,6,7,8].

Guava (*Psidium guajava* L.) is a sweet, aromatic fruit of the *Myrtaceae* family native from Central America [9]. This fruit is one of the most consumed fruits, and is of greater importance in the family basket of Latin American countries—especially Colombia, Mexico, and Brazil—as well as in some countries in Africa and Asia [9,10]. However, harvested guava can exhibit fast ripening during storage periods due to their high respiration rate and decay incidence [11,12,13,14,15]. Reported methods that can be applied to control the decay of fruits caused by microorganisms correspond to microbial antagonists, such as yeast, fungi and bacteria [16], chlorine dioxide, hydrogen peroxide, citric acid, and ethanol application [17], polysorbates [18], ozone (O_3_) [19], modified atmosphere packaging (MAP), ultraviolet-C (UV-C) light [20], electrolyzed water [21], gamma irradiation [22], and bioactive natural compounds such as essential oils [23,24], to avoid the use of synthetic fungicides in the control of fruit deterioration [25].

Essential oils (EO) are mixtures of strong odor with about 20 to 60 components including terpenes and terpenoids, coumarins and homologs of phenylpropanoids [26,27]. They could be an interesting method used in the delay of the deterioration of fruits. However, plant essential oils are volatile, and could potentially affect odor and flavor with the possible presence of phytotoxicity [28]. As a more recent strategy to preserve fruits in the post-harvest stage, the use of coatings based on emulsions of biopolymers and essential oils with strong antimicrobial activity were reported [29]. Thus, microbial growth on the fruit surface is inhibited [19], preserving the overall fruit quality, the nutritional composition and the acceptance of the product [29,30].

It has been identified that the genus *Ruta* contain several active compounds including coumarins, flavonoids, furanocoumarins, and alkaloids. Due to their chemical composition, several studies have indicated that *Ruta graveolens* (rue) has potent antimicrobial activity. Besides, topical pharmaceutical fungicides were prepared from rue extracts [31,32,33,34,35,36,37,38,39,40]. 

On the other hand, several antifungal compounds were found in *R. graveolens* [41]. The 5- and 8-methoxypsoralen, were tested against different fungi (Rhizoctonia solanii, Fusarium spp., Pyrenochaeta lycopersici, Trichoderma viride, Penicillium spp., Thielaviopsis basicola, and Verticillium dahliae) with interesting results [41,42,43]. 

*C. gloeosporioides* is a fungus that causes more loss in the world, recognized as the cause of anthracnose disease. This disease causes blackening and deterioration of the fruit, generating large economic losses to producers and throughout the value chain [29].

Otherwise, a deacetylated and highly abundant derivative of chitin in nature—chitosan—is a linear polysaccharide consisting of (1,4)-linked 2-amino-deoxy-β-d-glucan [44]. 

Thanks to its characteristics, it has been deeply investigated for many applications [45]. Between those, some studies proved that chitosan coatings improved the storability of several perishable fruits, such as strawberry [24,46], tomato [47], litchi [48], longan [49], peach [50], mango [51] and table grapes [25]. For example, Oliveira et al. demonstrated the antifungal effect of chitosan-*Mentha piperita* essential oil in situ and in vivo against different *Colletotrichum* species (*C. asianum*, *C. dianesei*, *C. fructicola*, *C. tropicale*, and *C. karstii*), responsible of anthracnose in mango (*Mangifera indica* L.) [52].

Bill et al. (2014) [53] observed a strong fungicidal effect after 10 days of inoculation in vitro of the mycelial growth of *C. gloeosporioides* using a combination of chitosan and *Thyme* essential oil. Nanostructured edible coatings of chitosan and *Thyme* essential oil were applied to improve the postharvest quality of avocado with excellent results in the control of *C. gloeosporioides* [54].

Chitosan coatings not only provide a microbial barrier, but also provide a barrier against moisture, the entry of oxygen, the loss of ethylene and preserve bioactive compounds against pathogenic microorganisms in food. In addition, it can retain antioxidant agents that contribute to the preservation of food for a longer time [55].

Previous studies have indicated that fresh-cut guava coated with chitosan improve physical-chemical properties—especially weight loss and maturity index [56]. Another study demonstrated positive changes in the physical-chemical of guava (*Psidium guajava* L.) fruit during cold storage properties using coatings containing 2.0% of chitosan and remarkably, changes in chlorophyll, malondialdehyde (MDA) and vitamin C contents during 12 days [15]. However, there is scarce literature that presents the impact of chitosan-essential oil coatings on the microbiological attributes, sensorial properties, and in situ inhibition of *C. gloesporioides* fungi onto guava.

To the best of our knowledge, CS+RGEO coatings have not been used to preserve the postharvest decay of red guava. We studied the effect of coatings on the physicochemical, microbiological, and organoleptic properties of guavas. The in situ effect on the growth of *C. gloesporioides* on guava was also evaluated. A remarkable inhibition of *C. gloesporioides* was demonstrated, as well as with a higher stability of the coated guavas with CS+RGEO, presumably by a synergistic effect of chitosan and RGEO, which could be a promising result for the application in food industry.

## 2. Materials and Methods

### 2.1. Composition of Essential Oil of Ruta Graveolens

The essential oil of *Ruta graveolens* was acquired from Krauters (Bogotá, Colombia), and its composition was determined by gas chromatography-mass spectrometry (GC-MS) according to the methodology reported by [57], using an AT 6890 Series plus gas chromatography spectrometer, with a mass selective detector (full scan). A DB-5MS fused silica capillary column was employed with a temperature ramp of 60 °C for 10 min, then 5 °C/min to 250 °C, and maintained for 10 min. The constituents were identified by comparing their RI (retention index) with those provided by Adams database (Wiley, 138 and NIST05, for Agilent, Santa Clara, California, CA, United States).

### 2.2. CS+RGEO Emulsion Preparation and Characterization 

The emulsions were prepared according to the procedure already reported by [30], incorporating to the 2% CS solutions (acetic acid 1%) RGEO (Sigma, Milwaukee, WI, USA) in different concentrations (0.5%, 1.0%, and 1.5% *v*/*v*, with respect to the chitosan solution).

#### 2.2.1. Particle Size 

Particle size of the CS+RGEO emulsions were tested as previously reported [24], an AIMSIZER 2011 laser diffractometer was used following the International Organization for Standardization [58].

#### 2.2.2. Viscosity Measurements

The viscosity was determined using a Brookfield LVF viscometer. The amount of moisture per sample was determined. Each sample was diluted in water at a temperature of 25 °C ± 0.2 °C in a beaker, placed under the agitator at low speed until the sample was homogeneous. The spin number, the Brookfield conversion factor and the speed in rpm were used according to the ASTM D2196-99 standard [59]. 

#### 2.2.3. Total Solid Content 

It was determined as the reported methodology [24], according to Equation (1):

(1) $$\%S=\left(\frac{P_{s}-P_{d}}{P_{m}-P_{d}}\right)\times 100$$

where *%S* is the percentage of non-volatile solids in the sample (*w*/*w*), *P_d_* is the weight of dry and clean aluminum disk (g), *P_m_* is the weight of the sample plus the aluminum disk (g), and *P_s_* is the weight of the dry sample plus the aluminum disc (g).

### 2.3. Treatments

In order to evaluate the effectiveness of the coatings, two different experiment were carried out. The first experiment aimed to evaluate the effectiveness of the CS+RGEO emulsions on the naturally contaminated fruit and the second experiment aimed to evaluate the efficacy of the emulsion to inhibit the *C. gloesporoides* growth in situ.

#### 2.3.1. Fruit Samples

Guavas were collected from a local producer (Jamundí, Valle del Cauca, Colombia) and selected according to the Colombian technical standard (NTC) 1263 related to the guava quality during the post-harvest stage (uniform size, length, color and shape, no mechanical damage or fungal detection, and weight).

#### 2.3.2. Evaluation of the Coatings on the Naturally Contaminated Fruits

Before coatings, guavas were washed with 0.5% (*v/v*) of CECURE^®^, a cetyl pyridinium chloride solution (Safe Foods, Rogers, AR, USA) and sterile distilled water. Four batches of 90 guavas each were left to dry at 25° C, and successively coated with the different treatments, which included: T1 = CS solution (chitosan 2% *w/v* in 1% acetic acid solution without RGEO), T2 = CS + RGEO 0.5%, T3 = CS + RGEO 1.0%, and T4 = CS + 1.5% RGEO—untreated samples were used as controls. Freshly prepared CS+RGEO emulsions were then sprayed uniformly on the guava’s surfaces. Successively, they were dried for one hour at a temperature of 24 °C ± 2 °C. The process was repeated to complete two layers of coating. The coated fruits were placed in expanded polypropylene (EPS) trays and stored at a temperature of 24 °C ± 2 °C and relative humidity of 70%, in a rack fitted with a protective mesh. Fifteen guavas of each treatment were sampled periodically to perform the different analysis.

#### 2.3.3. Evaluation of the Coatings on Inoculated Guavas

The fungus used in the trial corresponded to the highly pathogenic strain of *Colletotrichum gloeosporioides* isolated from yam plants. The inoculation of *C. gloesporoides* was performed by the colonized agar plug method proposed by Olivieira et al. [60] with some modifications. A disk (3 mm depth and 8 mm diameter) of guava tissue was removed using a sterile scalpel from three different sides of each fruit to be inoculated with the mycelia plugs from five day-old of *C. gloesporoides*. In order to guarantee the fungal colonization, inoculated guavas were placed in humid chambers (90% relative humidity, 25 °C ± 0.2 °C) made with self-closing polyethylene bags (ZIP), which on the inside contained sterile cotton impregnated with sterile deionized water (3 mL). The samples were stored in the humid chambers at a temperature of 25 °C ± 2. After that, the guavas were removed from the humid chambers and placed in expanded polypropylene (EPS) trays at the same conditions. successively, the CS + RGEO coatings (T1, T2, T3, and T4) were applied, and fruits were allowed to dry in an aseptic laminar flow cabinet for 2 h. Inoculated guavas without coating were used as the control. Three replicates of 15 guavas per treatment were used. The samples were stored at 25 °C for 12 days. The measures of the growth diameter of the fungus were recorded on days 6, 8, 10, and 12. The percentage of inhibition of growth was calculated with Equation (2).

(2) $$\%PWR=\frac{N-F}{N}\times 100,$$

where %PWR = percentage of wound reduction (%), N = control wound diameter (mm) and F = diameter of the sample wound with treatment (mm).

### 2.4. Quality Attributes of Guava Samples

#### 2.4.1. pH and Total Soluble Solids (TSS)

The determination of the pH was made using a Thermo-Fisher Scientific. The total soluble solids were determined with the aid of a Milwaukee MA871 refractometer at 21 °C. For both determinations, 25 g of the fruits were macerated and blended in an Oster Mod. 4655 and filtrated with a cotton canvas filter.

#### 2.4.2. Titratable Acidity

Five grams of fruits were blended in an Oster Mod. 4655 and homogenized with 50 mL of distilled water. The mixture was filtered (mesh 40) and titrated with 0.1N NaOH using phenolphthalein as indicator. The results were expressed as the percentage of citric acid, according to Equation (3).

(3) $$\%\;Citric\;acid=\frac{V_{1}\times N}{W}\times K\times 100,$$

where *V*_1_ is the volume of NaOH consumed (mL), W is the sample weight (g), *K* is the equivalent-weight of citric acid (0.064 g/meq), and N is the normality of NaOH (0.1 meq/mL).

#### 2.4.3. Maturation Index

The maturation index was calculated according to Equation (4):

(4) $$MI=\frac{\%BRIX}{\%ACID}$$

#### 2.4.4. Weight Loss

Weight loss was determined gravimetrically using Equation (5):

(5) $$\%Wl=\frac{\left(Wi-Wf\right)}{Wi}\times 100,$$

where *%Wl* is the percentage of weight loss, and *Wi* and *Wf*, are initial and final weight of each sample (g). 

#### 2.4.5. Water Activity (Aw)

The water activity was measured with an Aw Rotonic meter (mod HP23-A, HygroPalm, Bassersdorf, Switzerland), to determine the variation of the microbial growth potential in the epidermis of the fruits for each treatment. For this, circular portions of 3 cm in diameter were taken from the peel of each fruit—without pulp—and placed in the bottom of the sample holder of the equipment where the measurement was taken. Sampling was carried out in duplicate until the probe stabilized at a temperature of 24 °C ± 2 [46].

#### 2.4.6. Decay Index

**Figure 1:**
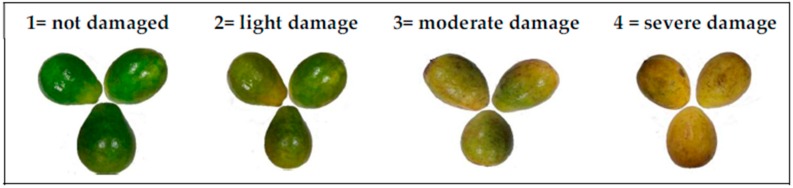
The damage scale of the guavas.

The decay index was evaluated utilizing the methodology used by Barrera et al. [61]. In the scale, the physical and mechanical deterioration caused by the presence of fungi at the epidermis of guavas, was visually evaluated using a damage scale from 1% to 20%, according to the following scale: 1 = not damaged; 2 = light damage (<10%); 3 = moderate damage (>10% and <20%); 4 = severe damage (> 20%). The results of the evaluation were expressed utilizing Equation (6):

(6) $$Decay\;index=\frac{1n+2n+3n+4n}{N},$$

where n = number of fruits classified in each level of the damage scale and N = number of total fruits analyzed in each treatment per day. The decay index of fruits was evaluated on days 0, 3, 6, 9, and 12 according to the scale in Figure 1.

#### 2.4.7. CO_2_ Respiration Rate

The equipment employed was an EcoChamber ME-6667 (PASCO, Roseville, California, CA, United States) including a carbon dioxide sensor PS-2110 used to measure the CO_2_ levels using gaseous CO_2_ analyzer during days 0, 3, 6, 9, and 12, of the coating process, according to [24].

#### 2.4.8. Firmness Analysis

Firmness was evaluated measuring the maximum force to penetrate the fruits, using cylindrical penetrometer (3 mm diameter) coupled with an EZ-Test (Shimadzu-USA) texturometer. A penetration speed of 5 mm/s was used. The penetration was made in three points on the equatorial zone of the fruits, and its average was reported.

#### 2.4.9. Color Parameters

The color of the surface of the fruits was determined using a colorimeter (CM-600d, Konica Minolta Optics Inc., Tokyo, Japan). CIELab color coordinates were obtained (L *, a *, and b *) using the D65 illuminant as a reference with an observer of 10 degrees. The range of the color parameters was L * = 0 (black) to 100 (white), a * = −60 (green) to +60 (red), and b * = −60 (blue) to +60 (yellow). The reported values correspond to the average of three measurements in each treatment. The measurements were made on the surface of the equatorial zone of the fruits at three random points.

### 2.5. Microbiological Analysis

#### 2.5.1. Yeast and Molds

Yeast and mold counts on the guavas surfaces were performed under ISO 7954 using the methodology reported elsewhere [24]. These analyses were carried out in triplicate, on days 0, 3, 6, 9, and 12 of the treatments. The results were reported as log UFC/g of molds and yeasts.

#### 2.5.2. Mesophylls Aerobic Counts

The mesophylls aerobic count was performed under ISO 4833 using the plate colony counting technique at 30 °C using the methodology previously reported [24]. The results were reported as logarithm colony-forming units per gram (log CFU/g) of mesophylls bacteria. 

### 2.6. Sensorial Analysis

The test was carried out considering the Colombian Technical Standard 3932 [24]. For the analysis, 50 untrained judges were required, and they were informed about the methodology of the test. They signed an informed consent which contained data on the reagents used in the preparation of the emulsions and the possible allergic reactions that they could experiment if they were sensitive. Each panelist had a time of approximately 10 min to answer all the sections of the format. In the test, the attributes of pulp color, flavor, aroma, texture, and brightness were evaluated for each of the samples, which were coded randomly. For the flavor sweep on the palate, they were informed that between sample and sample, they should eat a piece of salty biscuit and then drink water. The samples were evaluated, employing the hedonic scale of points consisted in nine levels: 1 = dislike extremely and 9 = like extremely. 

### 2.7. Statistical Analysis

The analysis of variance (ANOVA) and the Tukey method for mean separation, with a confidence level of 95% (α = 0.05), were used to evaluate the effect of edible coatings in the response variables described above. The Statgraphics Centurion XVI program was used for these statistical analyses. 

## 3. Results and Discussion

### 3.1. Essential Oil Characterization

The GC-MS analysis of the RGEO showed the presence of 48 compounds (Table 1). The compounds corresponded to 21 sesquiterpenes, four alcohols, seven ketones, five esters, one terpenoid, three sesquiterpenoids, two coumarins, and six non-identified compounds. Although terpenes and sesquiterpenes represent the main compounds in the great part of the essential oils, in RGEO ketones are the predominant compounds (76%), with 2-nonanone (23.5%) and 2-undecanone (42.6%)—hose in which the most relative abundance was detected. Our results are consistent with those reported in previous studies [62,63]. It is well known that the capability of the essential oils to inhibit fungal growth depend on their composition. In this context, the antifungal activity of the two main compounds of RGEO 2-nonanone and 2-undecanone has been reported [64]. However, it has been suggested that the presence of some antimicrobial constituents combined with other minor constitutes might be involved in improving the overall antimicrobial activity of volatile fractions [65,66]. Moreover, the synergy is not only influenced by the major compounds of EOs, since compounds present in low quantities may have an important role in this effect [67]. For example, Bassolé et al. reported the combination of eugenol with linalool or menthol exhibiting a higher antibacterial effect [68]. In this way, several binary or ternary combinations have exhibited synergistic antimicrobial activities for different mixtures of components of essential oils [69,70]. This synergy could be a result of some components present in EOs attached to the surface of the cell, and thereafter some other could penetrate the phospholipid bilayer of the cell membrane [57]. The result is a disruption of the cell membrane by their accumulation, negatively affecting the cell metabolism and causing cell death [71,72]. 

It is well known that around 80% of essential oils are constituted by one or two compounds, whereas other components are present in trace amounts [73]. Usually, the biological properties of the essential oils depend on major components that are classified into two different groups according to their origin—terpenes and terpenoids [74,75,76]. Enzymatic oxidation of terpene molecules results in the formation of terpenoid alcohols, ethers, ketones, and epoxides, such as Thymol, carvacrol, linalool, citronellal and some others [77]. It is very interesting that the precursor of carvacrol, *p*-cymene, a monoterpene with a benzene moiety without functional groups when is tested alone lacks of antimicrobial properties [78,79]. However, when is mixed with carvacrol the antimicrobial activity is increased [80]. 

Antimicrobial action of phenolic compounds such as thymol and carvacrol is attributed to structural and functional damages in the cytoplasmic membrane [81]. This increased antimicrobial effect is also observed when *p*-cymene is mixed with polymyxin B nona peptide [82]. This enhanced effect could be a result of *p*-cymene’s hydrophobic nature that causes swelling of the cytoplasmic membrane, creating channels that will allow cell’s penetration of more active compounds like carvacrol affect some internal organelles [83]. Also, *p*-cymene had an effect on the synthesis of protein in *E. coli* cells which could help with the antimicrobial effect [76]. Another possible explanation for the synergistic effect of several essential oil compounds could be that pathogens cannot acquire resistance to multiple components present in essential oils, for instance the action will remain effectively for longer periods [84]. Besides that, it has been reported that some essential oil components have the ability to decrease lipid enzymatic oxidation (LOX) by scavenging some oxidative radical species, decreasing the browning and deterioration of fruits [85]. It was also reported that although the activity of polyphenol oxidases (PPO), catalase (CAT), and peroxidases (POD) decreased over time for peaches, fruits that were packed inside active packaging with cinnamon essential oil, showed a lower decrease in these enzymes that are also related to the oxidative enzymatic browning of fruits, loss of sensory attributes, weight loss, and firmness. However, if the components of the essential oil have antioxidant capacity by means of radical scavenging, this enzymatic activity will be diminished by increasing the stability of the fruits, as could be the case of the CS + RGEO coatings whose major components are ketones with the ability to scavenging free radicals. If no deterioration is presented at the surface of the fruit, fungi will not be able to colonize and growth, extending the shelf-life of fruit.

### 3.2. Physicochemical Characterization of Chitosan Emulsions

For a successful film formation using the casting-film procedure, a stable emulsion should be prepared [86]. Table 2 shows the non-volatile fraction of the emulsion which is constituted by chitosan and the essential oil components presenting strong interactions. The coatings presented significant differences in the total solid content between CS treatment and those with RGEO content. Similar results were previously reported with CS and *Thymus capitatus* essential oil [24].

Normally, it is considered that analyzing the particle size of the emulsions, allows to determine the stability of the emulsion [24]. On the other hand, a smaller particle size could influence the physical and chemical properties such as the viscosity and density of the emulsions, due to the fact that a greater contact surface would be available that would improve the properties [87]. Chitosan helps with the stability of the emulsion as a colloidal protector by electrostatically adsorbing on the interface of the drop, preventing flocculation of the oil phase and the formation of cream [86]. It is considered that the observed effect of decreasing viscosity with the increase of the amount of essential oil in the emulsion, is the result of the reduction of the agglomeration of the oil phase thanks to the stability of the emulsion [86]. The same behavior of chitosan emulsions with essential oils of basil, thyme, bergamot, lemon, *Thymus capitatus,* and tea has been observed [24,88].

### 3.3. Physicochemical Analysis and Mechanical Properties of Coatings on Guava

#### 3.3.1. Changes in Titratable Acidity and pH

**Figure 2:**
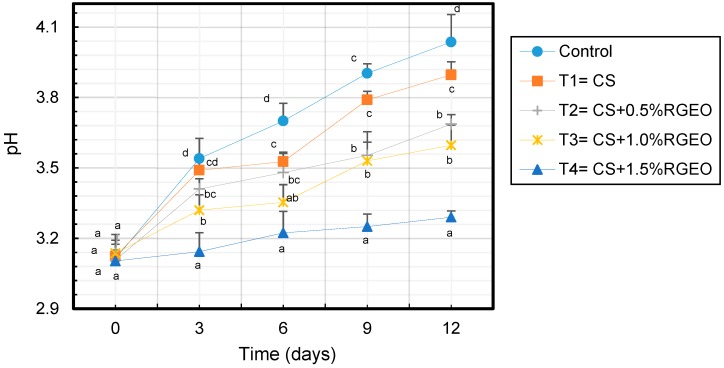
Evolution of pH in guavas with CS+RGEO treatments: Control = uncoated, T1 = CS, T2 = CS+RGEO 0.5%, T3 = CS+RGEO 1.0%, and T4 = CS+RGEO 1.5%. Mean values and intervals of Tukey’s 95% according to the ANOVA test. Different superscript letters in the same column indicate significant differences between treatments (a, b, c, d *= p* < 0.05).

Changes in pH values were registered during the storage time and significant differences (*p* < 0.05) were observed among the treatments (Figure 2). In fact, an increase of the pH was observed in all the treatments during the time which is related to the consumption of the high content of undissociated organic acids deposited in the vacuoles that fruits could use as respiratory substrate [89]. However, non-significant differences were evidenced between control samples and those coated with CS as they reached values of 3.90 ± 0.06 and 4.04 ± 0.11 at the end of the storage-time. However, samples coated with the emulsion of CS+RGEO were significant different. In particular, guavas coated with emulsions of 0.5% and 1.0% of RGEO reached values ranged between 3.69 ± 0.04 and 3.60 ± 0.09, while the samples coated with CS+1.5% of RGEO was of 3.29 ± 0.03. It is worth to mention that in this latter samples the pH decreasing was slowly, presumably due to the CS+RGEO coatings.

**Figure 3:**
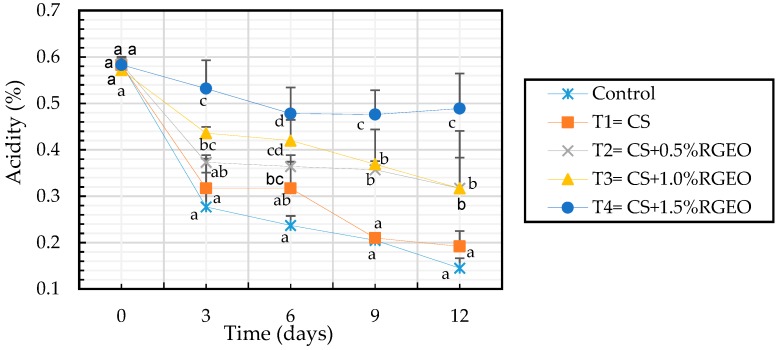
Evolution of the percentage of titratable acidity expressed as citric acid in guavas with CS+RGEO treatments: control, T1 = CS, T2 = CS+RGEO 0.5%, T3 = CS+RGEO 1.0%, and T4 = CS+RGEO 1.5. Mean values and intervals of Tukey’s 95% according to the ANOVA test. Different superscript letters in the same column indicate significant differences between treatments (a, b, c, d *= p* < 0.05).

It is well known that a reduction in acidity is related with the fruit maturation, accompanied of a sugar accumulation from starch degradation, while organic acids decrease since they are used as substrate for respiration process [90,91]. However, the role that organic acids in the ripening process is not completely understood [92]. Previous studies have demonstrated that chitosan coatings reduce the citric acid contents, which is the major organic acid in ripe guava fruit [93]. In our experiment, titratable acidity (TA) was reduced during the time in all the samples but with different reduction rates (Figure 3). Also, in this case the effect of the emulsions was significantly different with respect to the control and CS samples. In fact, and the end of the experimental period samples coated with CS+RGEO showed values ranged between 0.31% and 0.47% of citric acid, while control and CS samples showed values ranged between 0.145% and 0.192% of citric acid, respectively. Hong et al. 2012 [15], evidenced a retard in the loss of TA in guavas treated with CS coatings, effectively delaying fruit ripening. 

#### 3.3.2. Soluble Solids Content (SSC)

**Figure 4:**
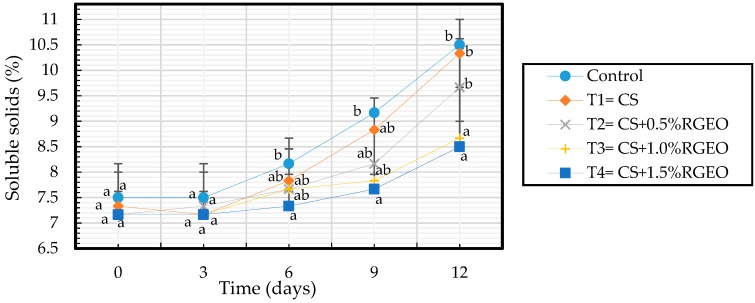
Evolution of the total soluble solids content by TSS measurement in guavas with CS+RGEO treatments: control, T1 = CS, T2 = CS+RGEO 0.5%, T3 = CS+RGEO 1.0%, and T4 = CS+RGEO 1.5. Mean values and intervals of Tukey’s 95% according to the ANOVA test. Different superscript letters in the same column indicate significant differences between treatments (a, b, c, d *= p* < 0.05).

Total soluble solids content increases with the ripening process due to the degradation of the starch caused by the increase of the activity of the hydrolases which gives rise the accumulation of sugars such as fructose, sucrose, and glucose [24]. However, as evidenced in Figure 4 the increase rate of SSC content was low in samples coated with CS+RGEO until day 9. This would happen if a modification of the internal atmosphere is produced, in which the concentration of the oxygen was reduced concomitant with an increase in the CO_2_ content suppressing the ethylene release as previously observed [24,94,95]. In addition, when O_2_ is not available, fruits degrade glucose anaerobically by glycolysis to generate energy. In the glycolysis pathway, aldehydes, alcohols, and lactates are produced causing an accumulation of anaerobic byproducts which produces off-flavors associated with physiological disorders, leading to an unacceptable eating quality [96,97]. This will explain at the beginning with low oxygen available for respiration, glucose is consumed anaerobically to produce energy. Then, CO_2_ will be produced retarding fruit ripening due to the inhibition of C_2_H_4_ [98]. The MA environment created around the fruit also reduced the respiration rates and the sensitivity of the fruit to C_2_H_4_ action [98].

#### 3.3.3. Maturity Index

**Figure 5:**
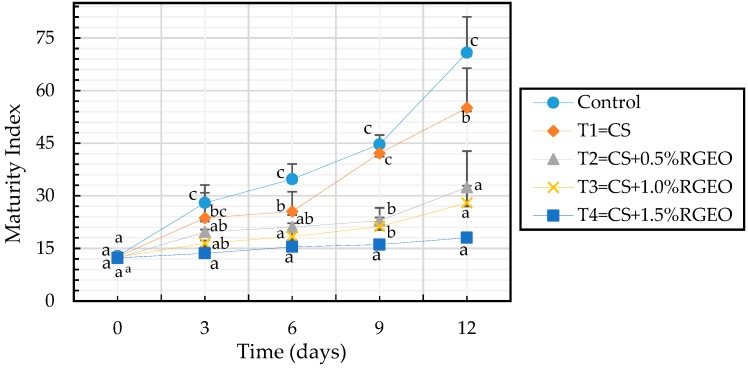
Maturity index of guavas during the storage time with CS+RGEO treatments: control, T1 = CS, T2 = CS+RGEO 0.5%, T3 = CS+RGEO 1.0%, and T4 = CS+RGEO 1.5%. Mean values and intervals of Tukey’s 95% according to the ANOVA test. Different superscript letters in the same column indicate significant differences between treatments (a, b, c, d *= p* < 0.05).

The maturity index is the ratio between the percentage of soluble solids and the percentage of acidity. Figure 5 shows that all treatments decreased the maturation index as compared to the control sample during the storage time, except for day 0. Also, after day 9 there were significant differences between the control sample, CS treatment, and emulsions of CS+RGEO but not RGEO concentration dependent. As stated above, in the maturation process of climacteric fruits such as guava, soluble solids (SSC) increase with time while the acids are consumed (decrease in acidity), increasing the pH [99].

When analyzing the previous figure it can be seen that the addition of RGEO with chitosan on the surface of the guavas as a coating generated a barrier effect that decreased the fruit ripening, presumably due to the interaction of the essential oil compounds with the components of the fruit, modifying the metabolism activity [95]. 

#### 3.3.4. Weight Loss and Water Activity

As observed in Table 3 all the samples showed weight losses with time. However, the coated samples showed lower loss than uncoated samples. On the other hand, there were small differences between the treatments with and without essential oil, indicating that the barrier to water loss was slightly influenced by the presence of the oil due to its hydrophobic effect. Normally, the unbound water migrates to the environment generating the weight loss [24]. As seen in Table 3, the guavas coated with CS+RGEO had a lower weight loss, so that an increase in the amount of oil allowed a lower loss of free water as will be discussed in the following section.

The change in water activity allows analyzing the behavior of free and bound water during fruit ripening, factors that directly influence the microbiological, physical, and chemical stability of food [100]. The results show a progressive decrease in the water activity concerning the storage days and the treatments applied. The statistical analysis indicated that there are significant differences (*p* < 0.05) in the storage time and between treatments. The control presented the most significant decrease going from 0.993 on day 0 to 0.963 on day 12. The treatments T3 and T4 showed the lowest reduction in Aw. The decay in water activity in the fruit is caused by the transformation of free water into bound water, responsible for transpiration reactions and hydrolysis during ripening [100]. This result agrees with the weight loss behavior of guavas with the treatments.

#### 3.3.5. Decay Index

It is well known that fungal growth on the guavas fruit surface under ambient conditions, decreased the fruits quality increasing the consumer rejection. In Colombia, anthracnose disease caused by *Colletotrichum* spp and “Roña” caused by *Pestalotia versicolor* are the principal diseases causing decay in guava fruit during crop, storage as well as ripening. However, the decay index is a visual parameter that allows to observe the physical evolution of fruits during storage, indicating the external damage on the skin.

**Figure 6:**
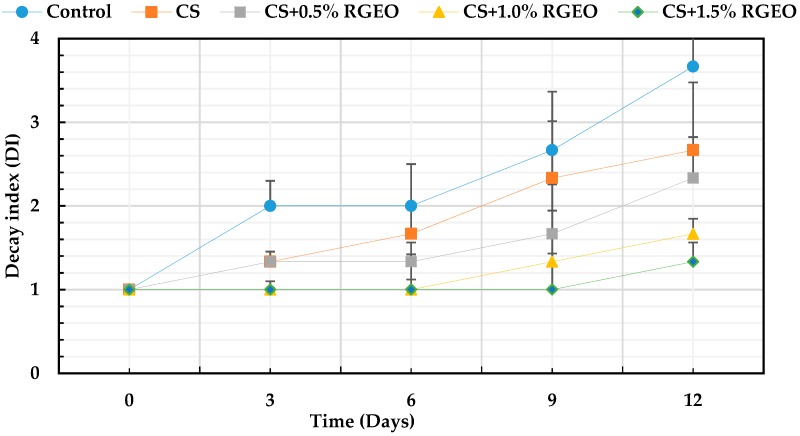
Evolution of the decay index in strawberries with chitosan (CS) and treatments of oil (TCEO): control, T1 = CS, T2 = CS+RGEO 0.5%, T3 = CS+RGEO 1.0%, and T4 = CS+RGEO 1.5%. Mean values and intervals of Tukey’s 95% according to the ANOVA test.

As can be seen in Figure 6, regardless of the treatment, the percentage of deterioration of naturally contaminated guavas increased with time. In particular, coated guavas deteriorated less during 12 days (from 2.3% to 1.3%) compared to the uncoated ones with significant differences (*p* < 0.05). It is evident that guavas treated only with CS reduced the decay index (21%). Additionally, guavas coated with CS+RGEO reduced the index decay (between 35% and 63%) compared to uncoated fruits. This reduction could be attributed to the coating barrier effect that lead inhibition of microbial growth, water loss as well as slow down of fruit ripening process. Different studies have demonstrated that fungi membrane mainly composed of fatty acids are affected by chitosan and some essential oil components [101]. Besides that, it is well known that the coatings of chitosan with essential oils generate a barrier effect to moisture, to the loss of ethylene, decreasing the rate of respiration and microbial attacks and therefore to the deterioration of the organoleptic properties [57].

Several investigations emphasize the capacity of the chitosan-essential oil coatings to diminish the fungal growth and the deterioration of the fruits [18]. In addition, edible films and coatings have been demonstrated contribute to decrease environmental troubles generated by conventional plastic packaging [18]. Mainly, there have been reports about the decrease of the attack and post-harvest losses caused by the fungi *Botrytis cinerea, Rhizopus stolonifer* and *Aspergillus niger* in strawberries and grapes. It should be noted that these are the main pathogens, together with the genus *Colletotrichum*, of fruits and vegetables [24,48,101]. Normally, the infection caused by these fungi deteriorates the fruit’s crust, causes browning, and varies the flavor and color of the fruits. The fungus *C. gloesporioides* is also the cause of anthracnose disease in fruits main responsible of several losses to farmers [54,56,66,102].

#### 3.3.6. CO_2_ Respiration Rate

**Figure 7:**
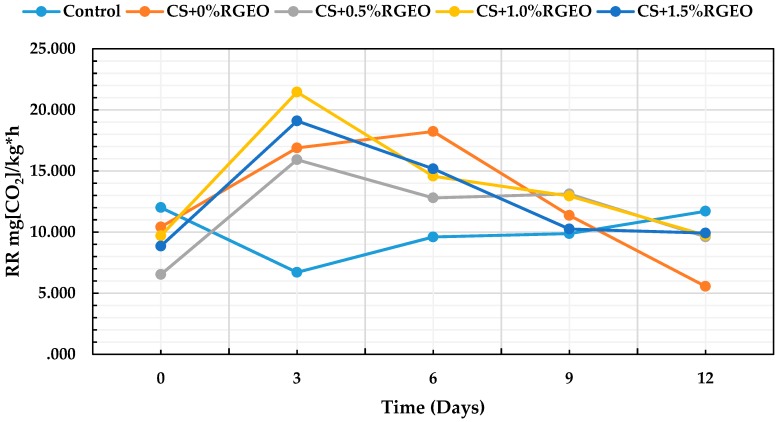
Evolution of CO_2_ respiration rate in guavas with CS+RGEO treatments: control, T1 = CS, T2 = CS+RGEO 0.5%, T3 = CS+RGEO 1.0%, and T4 = CS+RGEO 1.5%. Mean values and intervals of Tukey’s 95% according to the ANOVA test.

Guava can exhibit a fast ripening during storage periods due to their high respiration rate and decay incidence [15]. Figure 7 represents the values of the CO_2_ respiration rate, expressed as mg of CO_2_ kg^−1^h^−1^, on days 0, 3, 6, 9, and 12. 

The statistical analysis shows significant differences (*p* < 0.05) for the respiration rate between fruits with treatments and storage time (*p* < 0.05). From the previous image it can be shown that there were significant differences from day 0 in all treatments for each day. It has been documented that coatings of CH containing bergamot oil inhibited both O_2_ consumption and CO_2_ generation throughout the storage, which can be associated with lower gas permeability values of these films [30] only during the first eight storage days, which could be attributed to the progressive hydration of the film with the subsequent loss of the effect of the gas barrier, due to a strong interaction of chitosan and essential oil components. However, the hydration of the coating could explain why the barrier effect will decrease and the fruit will continue in its climacteric peak (between day 0 to day 6) due to the senescence. 

For the other side, modification of the gas environment will reduce respiration of the fruit producing lower CO_2_ as can be seen after day 6 for the fruits coated, a result that has been previously reported [103,104]. Reduction of the respiration rate will produce less ethylene, the phytohormone necessary for maturation trough metabolic pathways that use oxygen as well. For instance, a reduction in respiration will extend the shelf-life of the fruit. 

Usually, modified atmosphere packaging (MAP) storage and controlled atmosphere (CA) storage are used to increase the shelf life of fruits and vegetables [105]. If O_2_ concentration declines below the critical limit required for sustaining anaerobic respiration, fermentation will set in—resulting in the development of off-flavor [97,106]. On the other hand, the presence or the accumulation of high CO_2_ concentration could also have a negative effect on fruit quality by accelerating changes in color and firmness, and increasing the solubilization of pectic compounds [107]. However, from day 9, the coated fruits decreased their rate of CO_2_ production in comparison with the uncoated guavas. In another study, it was shown that chitosan treatments incorporating lemon essential oil decreased CO_2_ production compared to chitosan coatings at day 12. In our case, during day 12, guavas coated with chitosan showed less than those with CS + RGEO [95]. 

The natural process that occurs during fermentation—such as loss of aroma, flavor, texture, and fruit browning—can be controlled with coatings that reduce the respiration rate [88]. However, an excessive production of carbon dioxide could facilitate the fruit degradation thanks to the anaerobic fermentation produced by microorganisms with the consequent development of unpleasant flavors and aromas [24]. For this reason, it is necessary to carefully control with an expert sensory panel, any appearance of unpleasant colors or smells. In this investigation, no odors or flavors were detected, thanks to the barrier effect generated by the coatings of CS + RGEO against the decay of the fruit.

With these results, it is confirmed that the RGEO preserved the quality attributes of the guavas for a longer time, exerted a control of microorganisms that cause the deterioration of the fruit and, in addition, did not negatively affect the organoleptic properties of the guavas [86]. Different studies based on the application of chitosan coatings confirm the presented results, such as in the cases of apples [108], strawberries [109], and table grapes [110]. Thanks to the reduction of CO_2_ production, the sensory and quality attributes of guavas can be better retained without producing anaerobic fermentation within the fruit [99].

#### 3.3.7. Firmness Analysis

**Figure 8:**
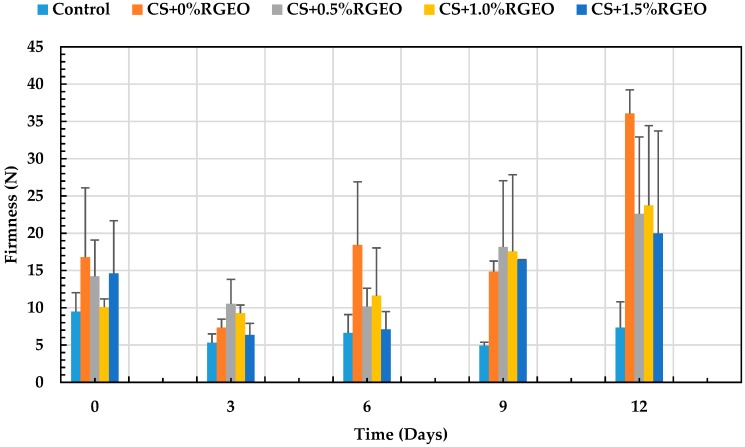
Evolution of firmness in guava with chitosan (CS) and oil treatments (RGEO): control, T1 = CS, T2 = CS+RGEO 0.5%, T3 = CS+RGEO 1.0%, and T4 = CS+RGEO 1.5%. Mean values and intervals of Tukey’s 95% according to the ANOVA test.

The accelerated loss of firmness that guava undergoes is a factor that fungi take advantage of during ripening, in order to colonize the fruit while degrading its sensory and quality attributes [15]. In storage, a decrease in firmness was observed, as shown in Figure 8. On day 12, the control recorded a minimum firmness of 7.34N. There were significant differences (*p* < 0.05) between the control and T1. During day 12, no significant differences between coatings with essential oil were observed. The best treatments on day 12 were T2, T3, and T4, with a firmness between 36.06 and 19.96N, respectively.

Barrera et al. [61] showed similar results, having a decrease in firmness more pronounced on day 3. The decline in the firmness in fruits is related to the presence of enzymes that degrade polymers such as pectins, which are responsible for the firmness of the fruit. However, it is possible that the components of the essential oil oxidize some enzymes involved with the deterioration of pectines, delaying the ripening process of the fruits and maintaining the firmness of the fruit for a more extended period [95]. Generally, it is believed that softening and deterioration is decreased due to a reduction in the activity of the cell wall hydrolases, such as polygalacturonase, pectin methylesterase and ß-galactosidase, as well as reduction in the solubilization and depolymerization of the cell wall pectin, induced by some components of the essential oil [97]. It has been well documented that chitosan-essential oils reduce transpiration and increase water retention, providing turgor to the fruit cells maintaining firmness [111,112,113]. The decreased firmness in fruits such as grapes is associated with the action of cell wall degrading enzymes that hydrolyze starch to sugar and protopectin to water-soluble pectin [111,114]. In addition, cell wall degrading enzymes are delivered by mold-forming fungi during colonization and infection, which induce the characteristic softening in infected fruits [30,115]. As a result, the chitosan-essential oil coatings can maintain firmness in fruits by reducing water loss and decreasing cell wall degradation through the deactivation of mold-forming fungi on infected fruits. In general, coating formulations that minimize weight loss are also better at maintaining firmness, since this attribute is highly influenced by water content [88]. This, combined with the decrease in C_2_H_4_ production, delayed the process of senescence, resulting in the retention of chlorophyll (green color) and textural quality (turgidity) of the fruit [98]. 

The presence in the uncoated fruits is a factor that causes the loss of moisture in the guavas as well. Therefore, the treatment with a more significant weight loss recorded the least firmness (control) and the treatment with less dehydration (T1) preserved more firmness. In this context, Hong et al., 2012 [15] suggested that the presence of CS coatings on the guava surface contributed to the maintenance of firmness in the guava treated with chitosan coating due to its ability to cover the cuticle and lenticels, thereby reducing respiration and other ripening process during storage. In some cases, it has been shown that the presence of essential oils could contribute to the loss of firmness as a consequence of the interaction of the cells with essential oil components that weaken their own interaction during the senescense process [30,101].

#### 3.3.8. Color Parameters

**Figure 9:**
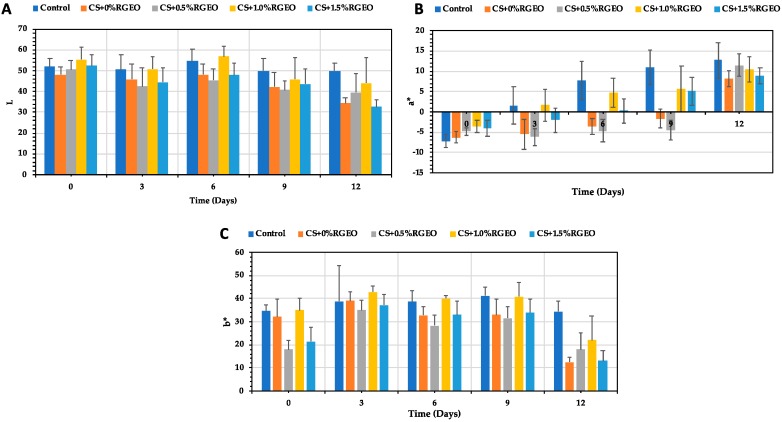
Color evolution during 12 days of guava with CS+RGEO treatments: control, T1 = CS, T2 = CS+RGEO 0.5%, T3 = CS+RGEO 1.0%, and T4 = CS+RGEO 1.5%. Mean values and intervals of Tukey’s 95% according to the ANOVA test. (**A**) Evolution of the L coordinate during 12 days with the CS+RGEO treatments. (**B**) Evolution of the a* coordinate during 12 days with the CS+RGEO treatments. (**C**) Evolution of the b* coordinate during 12 days with the CS+RGEO treatments.

Figure 9 shows the changes in the coordinates L, a-, and b- of guava samples with the time. The color is an attribute which influences the consumer in a decisive way, because it gives the idea of the degree of maturity that the fruit could have or could even generate in its rejection. According to the ANOVA test, there were significant differences (*p* < 0.05) between treatments and time of storage for the coordinate L (Figure 9A). All the treatments had a decrease in the L coordinate, indicating a browning of the tissues [88]. However, CS and CS+RGEO do not decline the decrease of the L coordinate. The L value was higher during days 0, 3, 6, and 9 but not 12 for T3 (CS+RGEO 1.0%). However, it was very close to the control values. L values decrease in all the treatments, including control, as a consequence of the release of water, which could be related to the darkening, as discussed earlier [102]. 

For the a* coordinate, all the treatments show a significant increase in the values, indicating a change in color from green to red (ripening). The difference was significantly higher for the control in all the days than for CS and CS+RGEO treatments. However, during days 3, 6, and 9, T1 and T2 had the lower values for the a* coordinate and during day 12, CS and CS+RGEO 1.5% (T4) had the lower a* coordinates, indicating a lower ripening process.

Finally, the b* coordinate decreased for all the treatments, but was significantly lower (*p* < 0.05) for CS and CS+RGEO 1.5%. The b* coordinate indicates the displacement from blue to yellow color. However, it was lower for coated than for control samples. An additional test of the coatings of CS + RGEO (0.5%, 1.0%, and 1.5%) controlled the maturation process showed that in the evaluated color scale a lower presence of yellow and red tones was observed in comparison with the uncoated samples. Similar results were previously obtained in other researches with chitosan-essential oil coatings [99,102].

### 3.4. Microbiological Analysis

As observed in Table 4, CS coatings have shown efficacy to inhibit the growth of the mesophilic bacteria, yeast and molds naturally present in the guava fruits. Although coatings CS and CS+RGEO 0.5% inhibited the bacteria growth for about 0.5 and 1.5 Log CFU/g after treatment, respectively, coatings with 1.0% and 1.5% of RGEO showed a stronger inhibition of about 3 Log CFU/g.

Throughout storage, there was an increase in the bacterial population growth in every treatment, even though guavas samples treated with 1.5% of RGEO showed significantly lower counts as compared to the control. Therefore, none of the treatments used in this experiment were able to eliminate mesophilic count on the fruit surface through storage; however, the growth of bacteria population was reduced significantly reaching of 1.5 log UFC/g in samples with T4. 

Although there are studies that demonstrate the efficacy of chitosan alone as an antifungal agent, the highest antifungal efficiency evidenced in this study was presented in guavas coated with CS+RGEO, suggesting the efficiency of RGEO to control fungi.

As it has been demonstrated previously, CS has a potent antifungal effect [29]. In addition, chitosan coatings incorporated with various essential oils inhibited or controlled the growth of fungal agents and allowed the extent of fruits stability time of storage [116]. In our work, a reduction of about 2 log CFU/g was observed after the treatment. This efficacy was intensifying (*p* < 0.05) by the addition of RGEO in the coatings. As can be observed in Table 4, after treatment and during the storage time, CS+RGEO coatings were more efficacious against fungi and yeast counts than against mesophilic bacteria, contributing to the extended shelf-life of the guavas. 

The statistical analysis indicated that there were significant differences (*p* < 0.05) during the storage time and between the treatments applied to guavas. In general, a decrease of the inhibitory effect of the CS+RGEO when the storage time increased was observed. In this context, de Oliveira et al. 2014 [60] suggested that during fruit ripening, the susceptibility to pathogenic fungi increases. In addition, it could be possible that RGEO volatilization contributes to decrease the efficacy of the emulsions.

### 3.5. Sensory Properties

**Figure 10:**
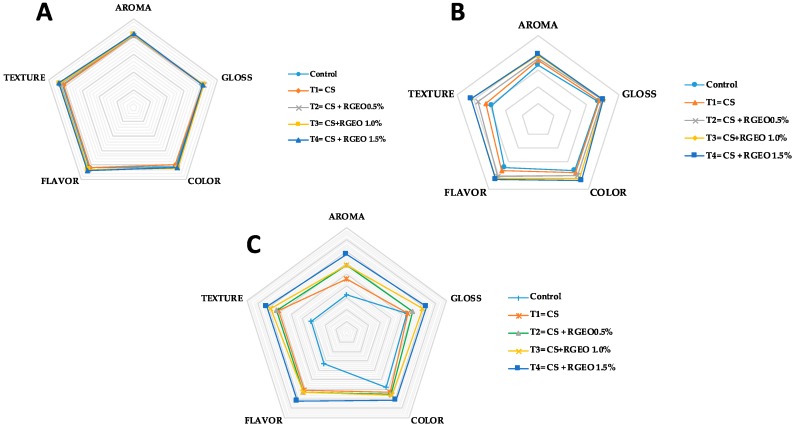
The hedonistic scale of guavas treated with CS+RGEO on days 0 (**A**); day 5 (**B**); and day 10 (**C).** Mean values and intervals of Tukey’s 95% according to the ANOVA test.

The sensory analysis of the coated samples is fundamental as it determines the acceptability of the final consumer of fruits. The results of the hedonic evaluations are observed in Figure 10 during day 0 (Figure 10A), day 5 (Figure 10B), and day 10 (Figure 10C). 

After treatment, sensory assessors did not observe differences between control and treated samples. However, significant differences on sensory attributes were detected by the untrained panelist during day five of storage. At this time, untreated samples were not accepted for the majority of the consumers, indicating that there was a major acceptability of the guavas treated with CS+RGEO 1.5% emulsion. This trend was observed to the end of the storage time as well. These results highlight that flavor, aroma, and texture were the attributes which had a major influence on the assessor’s acceptance, in line with non-physiological alteration of the guava fruits.

### 3.6. Antifungal Effects In Situ 

**Figure 11:**
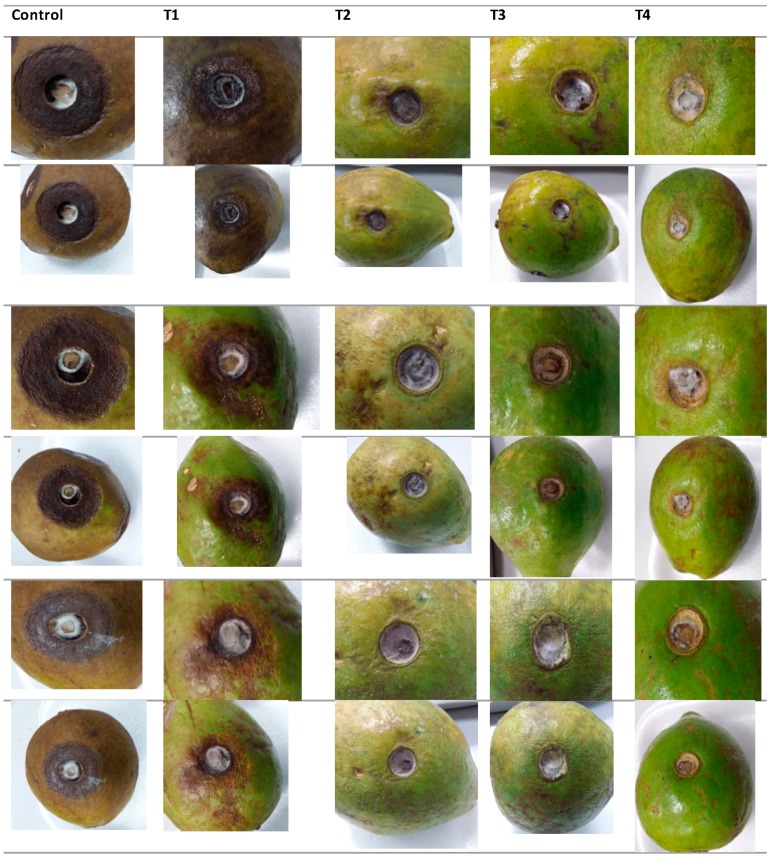
Images of growth inhibition of *C. gloesporoides* fungi in guava in situ inoculated at the end of the storage using CS+RGEO treatments: T1 = uncoated, T2 = CS, T3 = CS+RGEO 0.5%, T4 = CS+RGEO 1.0%, and T5 = CS+RGEO 1.5%.

The effects of chitosan emulsions applied on the inoculated guavas with *C. gloesporoides* are shown in Figure 11. Fruit sprayed with CS revealed a 41.36% of reduction in the fruit decay compared to untreated samples. The antifungal activity of chitosan considerably reducing the effect and development of the lesion caused by *C. gloesporoides* in fruits has been previously reported [29]. In the last years the activity of chitosan on fungal growth has been correlated to the expression of some enzymes in the fruits. For example, studies on mango cv. ‘Tommy Atkins’ fruit indicated that treatments of 1.0 % chitosan were able to induce gene expression of the defense enzymes peroxidase and polifenol oxidase in the fruit [117]. Recently, Obianom et al. 2019 [118] found that 1.5% of chitosan induced the up-regulation of the phenylalanine ammonia-lyase (PAL) gene and the down-regulation of lipoxygenase (LOX) gene, which could have contributed to improved anthracnose control. 

**Figure 12:**
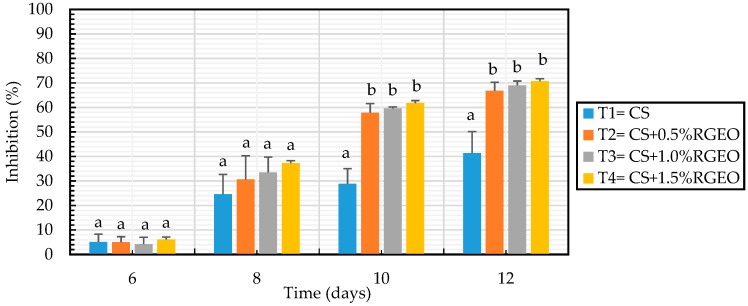
Growth inhibition of *C. gloesporoides* fungi in guava in situ inoculated using CS+RGEO treatments: control= uncoated, T1 = CS, T2 = CS+RGEO 0.5%, T3 = CS+RGEO 1.0%, and T4 = CS+RGEO 1.5%. Mean values and intervals of Tukey’s 95% according to the ANOVA test.

In general, the additive combinations between chitosan and RGEO show a high percentage of inhibition in the development of anthracnose lesions. Additionally, in this case, the increase of RGEO concentration significantly enhanced the inhibitory effects as they presented 66.82%, 68.98%, and 70.71% for the 0.5%, 1.0% and 1.5%, respectively (Figure 12). On the contrary, guavas fruit uncoated and treated with 0.1 M glacial acetic acid (pH 5.6), had 100% of fungal growth. There are few papers reporting the effect on CS-EO coatings against the growth of *C. gloesporoides* in situ. In this regard, Oliviera et al. (2012, 2018) [48] highlighted how combinations of chitosan and limonaria essential oil (*Cymbopogon citratus*) significantly decreased the diameter of the wounds in fruits inoculated with different phytopathogenic species of *Colletotrichum*. It could be possible that the effect of CS-EO coatings are due to the micropores structure of chitosan coating as the carrier and the antifungal activity of oil, which could reduce the respiration rate controlling the fungal decay of the fruit. However, Lima et al. [119] noted that the effect of inhibition on the growth of the fungus *Colletotrichum*—widely known to have a wide variety of host fruits—is not only influenced by antifungal treatments applied on the surface, but also depends on the physiology of the fruit making challenging to guarantee the same inhibitory result in different fruits. 

## 4. Conclusions

The results of the present study showed that the incorporation of *Ruta graveolens* essential oil into the edible chitosan coatings allows for the acquirement of stable emulsions of low viscosity and is easy to apply to the surface of the guavas. In addition, it was possible to reduce the growth of *C. gloesporioides* in guavas fruits, increasing their stability for at least 12 days at room temperature. In particular CS+RGEO 1.5% showed excellent stability concerning decay index, weight loss, maturation index, respiration rate, color, firmness, water activity, and microbiological decay as compared to the uncoated guavas and showed a high percentage of inhibition in the development of anthracnose lesions. This result could have potential application in the food industry. Future studies will be addressed to study the mechanisms of action of the CS+RGEO emulsions on the inhibition of *C gloesporoides*.

## Figures and Tables

**Table 1:** Volatile compounds identified in *Ruta graveolens* essential oil.

Chemical Function	Compound	RT	Amount Relative (%)	KI
Alcohol	2-undecanol	31.45	1.1	1304
	Manol	52.46	0.5	2076
	2-nonanol	23.84	3	1102
	1-nonanol	26.55	0.1	1172
Ketone	α-Thujone	24.25	0.1	1113
	2-undecanone	31.15	42.6	1296
	2-octanone	19.1	0.2	990
	2-decanone	27.38	4	1193
	(*R*)-(-)-Carvone	29.52	0.1	1251
	2-Dodecanone	34.93	2.9	1396
	2-nonanone	23.48	23.5	1094
	2-Tridecanone	38.44	2.5	1497
Ester	Octyl acetate	27.99	0.2	1209
	Benzyl acetate	46.24	1.7	1782
	1-Methylheptyl acetate	28.82	1.3	1232
	*trans*-farnesyl acetate	47.73	0.2	1834
	Benzyl 2-hydroxybenzoate	48.61	0.5	1887
Sesquiterpene	Nonyl acetate	31.62	0.7	1309
	Isodecanone	33.78	2.6	1366
	Geijerene	25.65	0.1	1149
	lsogeijerene C	29.98	0.1	1264
	Cogeijerene	30.36	0.2	1274
	Tetradecane	35.17	<0.1	1402
	*Cis*-β-Caryophyllene	35.7	0.1	1417
	Methyldecyl acetate	36.09	0.2	1429
	*trans*-β-Caryophyllene	36.28	0.8	1434
	(-)-Aromadendrene	36.53	0.9	1442
Sesquiterpene	Allo-aromadendrene	36.72	0.2	1447
	Isotridecanone	37.2	0.4	1461
	α-Humulene	37.53	1.1	1470
	γ-Muurolene	38.05	0.3	1485
	Geijerene	25.65	0.1	1149
	Valencene	38.64	0.2	1503
	α-Farnescene	38.75	0.2	1506
	γ-cadinene	39.31	0.2	1525
	σ-cadinene	39.41	0.5	1528
	α-Farnescene	43.44	0.2	1670
	(+)-cubenene	39.9	0.1	1545
Sesquiterpenoid	Viridiflorol	41.87	0.8	1611
	β-Eudesmol	43.52	0.2	1673
	Trans-Farnesol	44.72	0.3	1719
Furocoumarin	Ficusin	47.76	0.2	1849
	Chalepensin	54.8	1.1	2196
	N.I. (M+162)	29.76	0.9	1258
	N.I. (M+160)	43.61	0.3	1676
	N.I. (M+186)	43.7	1.1	1680
	N.I. (M+232)	47.25	1	1826
	N.I. (M+248)	51.94	0.4	2049
	N.I. (M+180)	52	0.1	2052

KI is the Kováts Retention Index relative to C5–C24 n-alkanes on the. DB-5 column.

**Table 2:** Physical properties of the CS+RGEO coatings.

Essential Oil (%)	pH	Density (g/mL)	Viscosity Brookfield (cP)	Solids (%)	Particle Size (μm)
0	4.38 ± 0.01 ^a^	1.0017 ± 0.01 ^a^	106 ± 0.1 ^d^	2.56 ± 0.02 ^a^	N.D.
0.5	4.40 ± 0.01 ^b^	1.0076 ± 0.01 ^a^	74 ± 0.1 ^c^	3.71 ± 0.01 ^b^	1.00 ± 0.25 ^a^
1.0	4.41 ± 0.01 ^c^	1.0080 ± 0.01 ^a^	66 ± 0.1 ^b^	3.87 ± 0.02 ^c^	1.22 ± 0.32 ^a^
1.5	4.43 ± 0.01 ^d^	1.0088 ± 0.01 ^a^	28.5 ± 0.2 ^a^	3.59 ± 0.02 ^d^	1.57 ± 0.12 ^a^

Values correspond to means ± standard deviation. Different superscript letters in the same column indicate significant differences between treatments (a, b, c, d *= p* < 0.05). N.D. = Not determined.

**Table 3:** Evolution of weight loss percentage and water activity evolution in guavas with CS+RGEO treatments: control, T1 = CS, T2 = CS+RGEO 0.5%, T3 = CS+RGEO 1.0%, and T4 = CS+RGEO 1.5%.

Days	0	3	6	9	12
**Weight loss (%)**					
Control	0	6.47	12.33	18.49	21.57
CS+0%RGEO	0	5.12	11.31	16.98	20.93
CS+0.5%RGEO	0	5.11	10.37	14.47	18.71
CS+1.0%RGEO	0	4.95	9.35	14.22	18.68
CS+1.5%RGEO	0	4.67	8.59	13.57	19.49
**Water activity**					
Control	0.993	0.984	0.975	0.965	0.967
CS+0%RGEO	0.975	0.967	0.965	0.96	0.951
CS+0.5%RGEO	0.978	0.976	0.971	0.965	0.946
CS+1.0%RGEO	0.985	0.97	0.969	0.963	0.956
CS+1.5%RGEO	0.977	0.974	0.971	0.96	0.957

Mean values and intervals of Tukey’s 95% according to the ANOVA test.

**Table 4:** Effect of treatments on the concentration of aerobic mesophilic, counting of molds and yeasts in guava with CS+RGEO treatments: Control = uncoated, T1 = CS, T2 = CS+RGEO 0.5%, T3 = CS+RGEO 1.0%, and T4 = CS+RGEO 1.5%.

Day	0			3			6			9			12		
	**Mesophilic bacteria (log UFC/g)**
Control	2.82	±	0.16 ^a^	4.11	±	0.19 ^a^	4.64	±	0.30 ^a^	5.18	±	0.15 ^a^	6.30	±	0.17 ^a^
T1 = CS	2.33	±	0.23 ^b^	3.02	±	0.12 ^b^	4.14	±	0.16 ^b^	5.00	±	0.14 ^b^	5.23	±	0.20 ^b^
T2 = CS+0.5%RGEO	1.50	±	0.09 ^c^	2.50	±	0.20 ^c^	3.26	±	0.08 ^c^	3.94	±	0.21 ^c^	4.34	±	0.10 ^c^
T3 = CS+1.0%RGEO	0.00	±	0.00 ^d^	0.00	±	0.00 ^d^	1.35	±	0.12 ^d^	2.26	±	0.14 ^d^	2.57	±	0.13 ^d^
T4 = CS+1.5%RGEO	0.00	±	0.00 ^d^	0.00	±	0.00 ^d^	0.00	±	0.00 ^d^	0.00	±	0.00 ^e^	1.35	±	0.12 ^e^
	**Yeast and Molds (Log UFC/g)**														
Control	2.73	±	0.13 ^a^	3.52	±	0.16 ^a^	4.10	±	0.12 ^a^	4.72	±	0.13 ^a^	5.99	±	0.27 ^a^
T1 = CS	0.95	±	0.12 ^b^	1.55	±	0.10 ^b^	1.63	±	0.23 ^b^	2.47	±	0.17 ^b^	2.57	±	0.14 ^b^
T2 = CS+0.5%RGEO	0.00	±	0.00 ^c^	0.00	±	0.00 ^c^	0.00	±	0.00 ^c^	0.00	±	0.00 ^c^	0.75	±	0.12 ^c^
T3 = CS+1.0%RGEO	0.00	±	0.00 ^c^	0.00	±	0.00 ^c^	0.00	±	0.00 ^c^	0.00	±	0.00 ^c^	0.00	±	0.00 ^c^
T4 = CS+1.5%RGEO	0.00	±	0.00 ^c^	0.00	±	0.00 ^c^	0.00	±	0.00 ^c^	0.00	±	0.00 ^c^	0.00	±	0.00 ^c^

Mean values and intervals of Tukey’s 95% according to the ANOVA test. Different superscript letters in the same column indicate significant differences between treatments (a, b, c, d = *p* < 0.05).
